# Relationship between physical activity, body mass index (BMI) and lipid profile of students in Ghana

**DOI:** 10.11604/pamj.2019.33.30.17889

**Published:** 2019-05-15

**Authors:** Eric Kwasi Ofori, Seth Kwadjo Angmorterh

**Affiliations:** 1 Department of Medical Imaging, School of Allied Health Sciences, University of Health and Allied Sciences, Ho, Ghana

**Keywords:** Physical activity, lipid profile, body mass index

## Abstract

**Introduction:**

In Ghana, there is no data regarding physical activity habits and lipid profiles of students. Therefore, the aim of this study was to investigate the relationship between physical activities, Body Mass Index (BMI) and lipid profile of students in Ghana.

**Methods:**

Cluster and systematic sampling techniques were employed to recruit 120 students, aged 18 years and above. This cross-sectional study was carried out among students from the University of Ghana. Biochemical analysis was conducted analysing total cholesterol (TC), high density lipoprotein (HDL), low density lipoprotein (LDL) and triglycerides (TG) in serum samples. Anthropometry measurements were also taken and BMI calculated. The physical activities, undertaken over a 7-day period, by the students were assessed using the International Physical Activity Questionnaire (IPAQ).

**Results:**

31.7% and 21.7% of the students were overweight and obese respectively. 61.5% of the obese students were engaged in high level physical activity as compared to 45.5% and 36.8% of the normal and overweight students, respectively. Normal weight students and overweight students showed significant differences in means of TC; [(4.56 ± 0.930 mmol/L) and (5.06 ± 0.93 mmol/L), respectively] and also between normal weight group (4.54 ± 0.93 mmol/L) and the obese students (5.24 ± 1.18 mmol/L). Significant correlations were also observed between TG, TC and BMI; and TC and TG, HDL and a strong correlation between LDL and TC (r=0.967).

**Conclusion:**

Strong correlations between BMI, physical activity and lipid profile indices among students in Ghana. Comprehensive efforts should be applied to reduce the incidence of CVDs among students.

## Introduction

Globally, the leading cause of death are non-communicable diseases (NCDs) [1]. These have impacted greatly on the burden of the management of diseases around the globe. The incidence of cardiovascular diseases (CVD), a NCD, is on the rise [2]. In Ghana, CVDs are the leading cause of death [3]. Lifestyle behaviours known to increase CVDs risks include high alcohol consumption, poor eating habits, physical inactivity, extended sitting time and smoking [4-6]. In Ghana, adolescents have poor eating habits; often eating nutritionally unbalanced and unhealthy diet that is low in dietary fibre and high in simple sugars, fats and sodium [7]. The onset of CVDs is also linked to serum concentrations of the various categories of lipids in the body. Higher levels of high density lipoproteins (HDL) and low levels of low density lipoproteins (LDL) are cardio protective [8]. Diets rich in saturated fats increase the serum concentration level of LDL, whereas the dietary approach to stop hypertension (DASH) diet which is rich in fruits, vegetables and monounsaturated fatty acids increases HDL levels thereby protecting the heart against CVDs [9]. In addition to the effect of diet on cardiovascular health, the level of physical activity is important in protecting against CVDs [10]. It is recommended that healthy adults engage in about 150 minutes of physical activity every week [11]. However, this cannot be said to be the norm among Ghanaians [12, 13]. University education can be stressful for students and can have negative impact on lifestyle behaviours such as poor eating habits and low physical activity [14]. This can increase the risk of CVDs. The American College Health Association reported an increase in the risk of developing CVDs among university students [15]. Anecdotally, one would have been expected that university students would be more active and engage in healthy lifestyles due to their educational background and the fact that they may be privy to the important link between high levels of physical activity and good health. This position is supported by a study that found that non-university students were found to have a greater risk of developing chronic diseases than those in university [16]. In Ghana, there is no data regarding physical activity habits and lipid profiles of students and their risk of developing CVDs. Therefore, the aim of this study was to investigate the relationship between physical activities, Body Mass Index (BMI) and lipid profile of students in Ghana in order to investigate the risk of developing CVDs among Ghanaian students.

## Methods

**Ethical considerations:** ethical clearance for the study was obtained from the Ethics and Protocol Review Committee (EPRC) of the School of Biomedical and Allied Health Sciences of the University of Ghana. All subjects provided information regarding the study and their consent sought before participating in the study. Participants' confidentiality and anonymity were protected.

**Study design and sampling:** this cross-sectional study was carried out among students from the University of Ghana (UG). The UG is located in the Greater Accra Region of Ghana with an estimated student population of over 38,000. The students included in this study were 18 years and above. Since the UG is a very large geographical area, a cluster sampling method was used because it is the most time-efficient and cost-efficient probability design for large geographical areas [17]. Cluster sampling technique ensures an increased level of accessibility of perspective sample group members [17]. Additionally, a systematic sampling method was used to add a degree of system or process into the random selection of subjects and also to ensure that the population was evenly sampled [17]. Going by the widely quoted Cohen's effect size classification for one-way between groups analysis of variance (ANOVA), an expected large effect size will correspond to an effect size of 0.49 or more [18]. Hence a power analysis using the GPower computer software was conducted. The GPower software has been shown to have excellent accuracy and has been used in sample size calculations for many studies [19, 20]. The results from the power analysis conducted using the Gpower software showed that a sample of 120 volunteers would be needed to determine a large effect, 0.49 with 80% power, using one way repeated measures ANOVA between means with alpha at 0.05.

**Data collection:** socio-demographic information of the students was obtained by the use of a self-administered semi-structured questionnaire. Information obtained included their ages, programmes of study and their marital status. The data for biochemical analysis, anthropometry measurements and assessment of physical activity were taken by the authors with the assistance of two experienced biomedical scientist with over ten years work experience between them.

**Biochemical analysis:** three (3ml) of venipuncture blood was drawn from students into gel separator tubes. These were then centrifuged at 3000 rpm for 10 minutes and stored in Eppendorf tubes at -20ºC. The Vitros 5, IFS Chemistry Analyzer (New York, USA) was employed in analysing the total cholesterol, high density lipoprotein and triglycerides in the serum samples. The equation [LDL-chol] = [Total chol] - [HDL-chol] - ([TG]/2.2) adopted from a study [21] was used in estimating the Low density lipoprotein cholesterol (LDL) levels from the serum samples.

Anthropometry: height and weight measurements were taken, and BMI calculated and categorised based on WHO [22] recommendations; [(< 18.5kg/m^2^ -underweight; 18.5-24.9 kg/m^2^ -normal; 25.0-29.9 kg/m^2^ - overweight; ≥30.0 kg/m^2^ - obese)]. Weight was measured in kilogram (kg) with the HBF-516 body composition monitor and scale (Illinois, USA). The Seca stadiometer (Hamburg, Germany) was used to measure the height of the students in centimeters (cm).

**Assessment of physical activity:** students were asked about their engagement in physical activities. The international physical activity questionnaire (IPAQ) was used to assess the level of physical activity by recalling physical activities undertaken over a 7-day period. The recall assessed the type, duration and intensity of the activity taken. The activities were then categorised based on intensity and values were awarded based on metabolic equivalents (METS). Light activities were assigned an average of 1.5 METS, moderate activities 4 METS and vigorous activities were assigned 8 METS. The activity score was calculated by multiplying the minutes spent in each activity by the number of days of the activity. The average MET for the category was calculated and summed over all categories. Total physical activity score was then classified into high (≥3000 METSMin/Week), moderate (600-2999 METSMin/Week) and low (<600 METSMin/Week).

**Statistical analysis:** statistical analysis was conducted using SPSS v17. Means and standard deviations (SD) were used to summarise continuous data and frequencies and percentages used for categorical variables. Prior to conducting inferential statistics, the data was assessed for normality subjectively using histograms and stem and leaf and objectively using the Kolmogorov-Smirnov test. The results of normality testing indicated that the data was normally distributed, Kolmogorov-Smirnov test indicated nonstatistically significant p- values (p≥0.05). Consequently, hence parametric tests were conducted. The independent samples t-tests were used to test for differences between the means of two groups and one-way between groups ANOVA to test for statistically significant differences between three or more groups. Post-hoc analysis was conducted using the Bonferroni adjustment confidence interval (CI). Pearson's correlation was conducted to assess the relationship between variables. In all cases, statistically significance level was set at p≤0.05.

## Results

**Demographics:** the study involved 120 students. The mean age of the students was 30 ± 7.9 years. Chi square tests were conducted to established relationships between the variables and gender. The results are presented in Table 1. The lipid profile of the students is presented in Table 2. With the exception of HDL, no significant difference was observed among the males and females students. The female students had significantly higher levels of HDL (1.46 ± 0.25) compared to the males (1.20 ± 0.22). The BMI of the students were classified according to international standard [22]. The results from this study indicated that 31.7 and 21.7% of the students were overweight and obese respectively. The lipid profiles of the students were assessed according to their BMI classification (Table 3). The parametric analysis of variance (ANOVA) between groups was carried out between the normal, overweight and obese groups. The underweight group was excluded from the ANOVA tests because only one student was classified as underweight. The analysis was carried out to compare lipid profile in TG, TC, HDL and LDL levels among the different BMI categories. The results showed that there was a statistically significant effect of BMI on TG levels for all three BMI categories (p=0.001). Also, statistically significant effects was also observed of BMI and TC levels (p=0.005), HDL levels (p=0.018) and LDL levels (p=0.04). Due to the significant effects shown, a post hoc analysis was carried out using the Bonferroni adjustment Confidence Interval (CI). Post-hoc analysis indicated that TG levels for the obese group (1.06 ± 0.44) was different from both the overweight (0.77 ± 0.26) and normal weight groups (0.75 ± 0.29); having statistically significant p-value (p=0.001). However, there was no statistically significant difference between the overweight (0.77 ± 0.26) and normal weight group (0.75 ± 0.29); p=0.09. In the case of TC levels, the difference observed was in the means of normal weight group (4.54 ± 0.93) mmol/L and overweight group (5.06 ± 0.93) mmol/L; and then between the normal weight group (4.54 ± 0.93) mmol/L and the obese students (5.24 ± 1.18) mmol/L; all having statistically significant p-values (p=0.001). The difference in the means of the groups in terms of HDL levels was observed only between normal weight students (1.36 ± 0.24) mmol/L and the overweight students (1.51 ± 0.28) mmol/L; p=0.001. Table 4 presents the physical activity levels of the students according to their BMI categories. The results indicate that 61.5% of the obese students engaged in high level physical activity as compared to the normal (45.5%) and overweight (36.8%) students. More of the overweight students (57.9%) engaged in moderate physical activities compared to their other counterparts. Correlations between the different variables tested are presented in Table 5. Strong, significant and positive correlation was observed between LDL and TC and negative significant low correlations was also seen between HDL and TG. It is also important to note that there were negative correlations between LDL and Physical activity and TG and physical activity level even though the test was not significant.

**Table 1:** results of chi square tests comparing the demographics between male and female students

Variable	Male (n=20)	Female (n=100)	Total (n=120)	p-value
Age in years (Mean±SD*)	26.65±5.69	30.72±8.23	30.04±7.99	0.037
Anthropometry (Mean±SD) Height (cm)	170.31±5.73	162.68±5.91	163.95±6.52	0.001
Weight (kg)	64.22±9.83	72.30±16.94	70.96±16.22	0.042
BMI(kg/m2)	22.08±3.52	27.22±5.24	26.37±5.71	0.001

* SD= standard deviation

**Table 2:** comparison of lipid profile according to gender (n=120)

Lipid	Male (n=20)	Female (n=120)	Total (n=120)	p-value
TG	0.90±0.39	0.81±0.33	0.82±0.34	0.258
TC	4.58±1.08	4.91±1.01	4.86±1.03	0.189
HDL	1.20±0.22	1.46±0.25	1.42±0.27	0.001
LDL	2.97±1.11	3.08±0.94	3.06±0.96	0.637

*SD= standard deviation

**Table 3:** lipid profile of students according to BMI classification (N=120)

Lipid profile (mmol/l)	Underweight (n=1)	Normal (n=55)	Overweight (n=38)	Obese (n=26)
TG	0.98	0.74±0.30	0.77±0.26	1.06±0.44
TC	3.64	4.56±0.93	5.06±0.93	5.23±1.18
HDL	1.40	1.36±0.24	1.52±0.28	1.40±0.26
LDL	1.79	2.86±0.89	3.20±0.92	3.35±1.09

**Table 4:** physical activity level of students according to BMI categorization (N=120)

Physical activity level	Underweight n (%)	Normal n (%)	Overweight n (%)	Obese n (%)
Low	1 ( 100.0)	4 (7.3)	2 (5.3)	1 (3.8)
Moderate	0 (0.0)	26 (47.3)	22 (57.9)	9 (34.6)
High	0 (0.0)	25 (45.5)	14 (36.8)	16 (61.5)
Total	1 (100.0)	55 (100.0)	38 (100.0)	26 (100.0)

**Table 5:** correlations between BMI, TG, TC, HDL and physical activity level of students

Variables	1	2	3	4	5	6	7
1. Body Mass Index of students							
2. Level of Triglycerides (mmol/l)	0.420						
3. Level of cholesterol (mmol/l)	0.215	0.259					
4. HDL Cholesterol (mmol/l)	0.107	-0.314	0.209				
5. LDL Cholesterol (mmol/l)	0.131	0.201	0.967	-0.002			
6. Physical activity level (MET-minutes/week)	0.099	-0.082	-0.103	-0.084	-0.074		
7. Age (years)	0.454	0.217	0.103	-0.010	0.072	0.084	

## Discussion

Physical activity, BMI and lipid profile are important indicators of an individual's cardiovascular health [23]. Regular physical activity and BMI within acceptable ranges are cardio protective and have an enormous positive impact in reducing the risk of CVDs [24]. Also, blood concentrations of the various lipids also provide important information on an individual's cardiovascular health [25]. Therefore undertaking a research to investigate the relationship between BMI and lipid profile among students in Ghana will provide a baseline data in addressing the high incidence rate of CVDs in Ghana. The study reported majority of the obese students (61.5%) engaged in high level physical activity compared to the rest of the categories. This finding is not surprising because obese students may be motivated to indulge in high levels of physical activity in order to lose weight compared to the rest. However a study indicated that obesity rather leads to physical inactivity [26]. Earlier studies suggested that high BMI is strongly associated with cardiovascular biomarkers rather than physical inactivity [27]. Furthermore, a study conducted by Dudina [28] postulated that any 5-unit increase in BMI was associated with increased CVD risk. The results obtained in this study contrasts the results of a study [29] carried out among Ghanaian civil servants in which high levels of physical activity was rather observed in only 3.4% of those who were obese and 4.3% in those who were overweight. The contradiction between the results of this study and previous studies may be due to the method used to assess physical activity. The international physical activity questionnaire (IPAQ) relied on self-reported data, rather than more objective methods for assessing physical activity, and this method of physical activity assessment is subject to over-reporting which may well be the case with the obese group. This is further supported by the fact that physical activity data showed no significant association with any measure of blood lipids which is contrary to much published research.

A recent study have showed associations between BMI and lipid profile indices in obese people [30]. Greater level of obesity which is exhibited in high BMI has also been linked to higher TC and lower HDL levels [31]. Post hoc test revealed significant differences between TG levels of overweight, obese and normal weight students. Significant differences were also observed in TC levels between normal weight students and overweight students. In addition, differences were also seen between normal weight students and obese students. This trend is contrary to another study [32] where TC levels did not show significant differences across the BMI categories. BMI also correlated positively and significantly with TG, TC and age. The trend observed in this study is in agreement with studies carried out among male college students from Riyadh where positive correlations existed between BMI and TC and LDL and negative correlation between BMI and HDL [32]. This occurrence supports earlier findings of high BMI, TG, TC and LDL levels correlating with each other and increasing the risk of CVDs [31]. It is also an established fact that abnormal levels of the various lipid profile indices have been linked to CVDs. This study reports significant increases in TG levels in overweight and obese students as compared to normal weight students. Similarly TC and LDL levels recorded similar observations. Although the trend is similar another study [33] where abnormal lipid profile were related to obesity, it should not be encouraged as it increases the risk of the onset of CVDs.

## Conclusion

In conclusion, the results of this study showed correlations between BMI, physical activity and lipid profile indices among students in Ghana. Comprehensive efforts should be applied to reduce the incidence of CVDs among students.

### What is known about this topic

- No study conducted on this topic in Ghana;
- No data available indicating the relationship between BMI, physical activity and lipid profile among students in Ghana.

### What this study adds

- This study shows that correlations exist between BMI, physical activity and lipid profile indices among university students in Ghana;
- Provides baseline data on the relationship between BMI, physical activity and lipid profile indices among students in Ghana.

## Competing interests

The authors declare no competing interests.
